# Human MDSCs derived from the bone marrow maintain their functional ability but have a reduced frequency of induction in the elderly compared to pediatric donors

**DOI:** 10.1186/s12979-020-00199-5

**Published:** 2020-09-09

**Authors:** Sara Magri, Elena Masetto, Samantha Solito, Samuela Francescato, Elisa Belluzzi, Assunta Pozzuoli, Antonio Berizzi, Pietro Ruggieri, Susanna Mandruzzato

**Affiliations:** 1 Department of Surgery, Oncology and Gastroenterology, University of Padova, Via Gattamelata, 64, 35128 Padova, Italy; 2 IOV-IRCCS, Padova, Italy; 3 Present address: University of Verona, Verona, Italy; 4 Pediatric Onco-Hematology Unit, Department of Women’s and Children’s Health, University of Padova, Padova, Italy; 5 Orthopedic and Traumatologic Clinic, Azienda Ospedaliera di Padova, Padova, Italy

**Keywords:** MDSCs, Aging, Immunosuppression

## Abstract

Myeloid-derived suppressor cells (MDSCs) are a heterogeneous population of immunosuppressive cells developing from myeloid progenitors, which are enriched in pathological conditions such as cancer, and are known to inhibit the functions of effector T cells. During aging, several changes occur both at the adaptive and innate immune system level, in a process defined as immunoscenescence. In particular, the low-grade inflammation state observed in the elderly appears to affect hematopoiesis. We previously demonstrated that the combination of GM-CSF and G-CSF drives the in vitro generation of bone marrow-derived MDSCs (BM-MDSCs) from precursors present in human bone marrow aspirates of healthy donors, and that these cells are endowed with a strong immune suppressive ability, resembling that of cancer-associated MDSCs. In the present work we investigated BM-MDSCs induction and functional ability in a cohort of pediatric versus elderly donors. To this aim, we analyzed the differences in maturation stages and ability to suppress T cell proliferation. We found that the ex vivo distribution of myeloid progenitors is similar between pediatric and elderly individuals, whereas after cytokine treatment a significant reduction in the more immature compartment is observed in the elderly. Despite the decreased frequency, BM-MDSCs maintain their suppressive capacity in aged donors. Taken together, these results indicate that in vitro induction of MDSCs from the BM is reduced with aging and opens new hypotheses on the role of age-related processes in myelopoiesis.

## Background

Myeloid-derived suppressor cells (MDSCs) are a heterogeneous group of immunosuppressive myeloid cells developing from myeloid progenitors, which are particularly enriched in pathological conditions such as cancer, infections, inflammation and sepsis [1–3]. These pathological conditions determine an increase in circulating colony-stimulating factors (CSFs) as well as chemokines that stimulate the myelopoiesis and, consequently, the generation of immature MDSCs. Different subsets of human MDSCs have been documented in several types of tumors, and it appears that all MDSC phenotypes can be allocated to one of the three main subsets, each of them containing more than one cell population. Monocytic MDSCs (M-MDSCs) share the morphology of monocytes and are characterized by the expression of CD14 and lack of CD15, polymorphonuclear MDSCs (PMN-MDSCs) are instead defined by the opposite expression of the myeloid markers (CD15^+^/CD14^−^), while more immature MDSCs (early-stage, e-MDSCs) lack the expression of both markers [4].

These cells are released into the bloodstream and recruited to the affected tissues, where they proliferate and are activated by inflammatory factors, and suppress acute inflammatory reactions by inhibiting the functions of distinct components of innate and adaptive immunity [5]. In tumors and inflammatory disorders T cells represent the main target of MDSC-induced immune tolerance [1, 6]. Indeed, MDSCs are involved in tumor angiogenesis, drug resistance and tumor progression and could represent a potential therapeutic target both in cancer and in chronic inflammatory diseases [3, 7].

During aging several changes take place such as the dysregulation of the immune, central and peripheral nervous, endocrine and metabolic system. In particular, aging is associated with a decline of functional capacity of both the adaptive and innate immune systems, in a process defined as immunoscenescence. Although the adaptive immune response has been more extensively investigated, some works document the significant impact of ageing on the innate compartment as well [6, 8, 9]. In fact, neutrophils, dendritic cells and monocytes in aged individuals show a reduced functional activity [10–13]. In addition, during aging an increase of regulatory T cells, a loss of T helper CD4^+^ and cytotoxic CD8^+^ T cells and an alteration of their functional capacities is observed, probably due to lower production of lymphoid cells by the bone marrow and to thymic involution, which reduces the release of naïve T cells. This is accompanied by a number of events including a reduced proliferative ability upon stimuli, as well as telomeres erosion, accumulation of memory T cells from chronic infections and replicative senescence upon persistent antigen exposure [14–16]. In the same context MDSCs accumulate and exacerbate the process by impairing T cell proliferation and function, and producing large amounts of pro-inflammatory cytokines [5].

Aging is also associated with a chronic, low-grade inflammation state called inflammaging [17]. It appears that in the microenvironment of the bone marrow (BM) inflammaging affects haematopoietic stem cells, with a possible rebound on the myelopoiesis and lymphopoiesis process [18]. In particular, one of the hallmarks of the alterations in the BM during aging is the increased myelopoiesis, associated with a concomitant decrease in lymphopoiesis.

Our group demonstrated that GM-CSF, G-CSF, and IL-6 allow the in vitro generation of MDSCs from precursors present in human bone marrow aspirates of healthy donors, and named such cells BM-derived MDSCs (BM-MDSCs). Of note, these cells share the phenotype and the suppressive function of MDSCs isolated from cancer patients [19]. BM-MDSCs are a heterogeneous collection of immature myeloid cells, but only the most immature subset from BM-MDSCs, with morphology and phenotype of promyelocytes (immature-BM-MDSC, i-BM-MDSC) is entirely responsible for the suppression mediated by BM-MDSCs [19].

Most of the works that advance that MDSC levels increase during aging have been obtained in mouse models in which these suppressive populations have been evaluated in the BM, spleen and circulation [20, 21], while only a few works have documented it also in humans [22–24].

It is not clear if the increase of myelopoiesis, which occurs in aging, is associated with an increase of MDSCs generation in the BM of humans, and consequently with an increase of these cells in the blood circulation. In addition, there are no data showing whether aging impacts on the immunosuppressive ability of MDSCs.

Aim of this study is to compare the induction of MDSCs from the BM of young and old individuals by using an optimized method to generate in 4 days MDSCs from precursor cells through cytokines treatment. Such cells are equivalent to MDSCs present in the blood of cancer patients and gives us the possibility to evaluate not only the expansion ability of the precursors, but also the immunosuppressive ability of the induced myeloid suppressor cells.

## Results and discussion

**Fig. 1:**
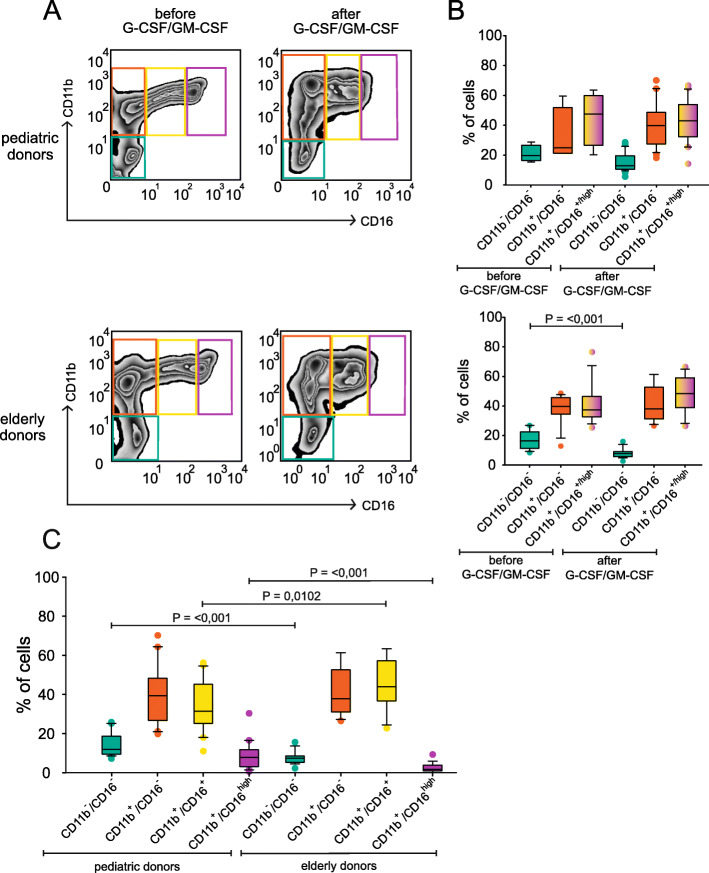
Human MDSCs are induced from BM precursors of pediatric and elderly donors by growth factors. **a** Representative flow cytometry with CD11b and CD16 on BM cells before (left panels) and after (right panels) 4 days of cell culture with growth factors G-CSF and GM-CSF. Upper panels, pediatric BM cells; bottom panels, BM from elderly donors. **b** Percentage of the different myeloid differentiating subsets among BM cells (*n* = 4 independent experiments) or BM cultures (*n* = 20 independent experiments) for pediatric patients (upper panel), and percentage of the different myeloid differentiating subsets among BM cells (*n* = 14) or BM cultures (*n* = 18) for elderly patients (bottom panel). **c** Percentage of the different myeloid differentiating subsets after four days of cell culture with growth factors between pediatric and elderly donors. Mann-Whitney U test was performed according to data distribution

In this study a total of 20 pediatric and 24 elderly donors were enrolled. Demographic data are reported in Table 1, showing a median age of 6 years for pediatric donors, and of 79 years for elderly individuals. BM aspirates from both pediatric and elderly donors with normal cytologic characteristics were freshly characterized for the myeloid differentiation by flow cytometry using by combining the expression of the markers CD11b and CD16. To induce the expansion of BM-MDSCs from myeloid cell precursors, we cultured BM cells for 4 days with the combination of the cytokines G-CSF and GM-CSF, and assessed the maturation of the myelomonocytic precursors by flow cytometry, as previously reported [19, 25]. A representative example of the immunophenotype of a BM before and after cell culture of a pediatric and an adult individual is shown in Fig. 1a, while the cumulative result of several independent experiments is shown in Fig. 1b. Three different myeloid subsets were analyzed: the most immature and immune suppressive fraction corresponding to CD11b^low/−^/CD16^−^ (immature-BM-MDSCs, i-BM-MDSCs), and the two more differentiated but immature and non-suppressive CD11b^+^/CD16^−^ and CD11^+^/CD16^+^ subsets [19] (Fig. 1 a-b).

**Table 1:** Characteristics of healthy volunteers

Variable	Pediatric (20)	Elderly (24)	***P***-value
**Age (year)**	6	79	< 0.0001
**Median [interquartile range]**	[7–4]	[82–76]	
**Sex**			0.952
**Female (%)**	6 (30)	7 (29.2)	
**Male (%)**	14 (70)	17 (70.8)

When we compared the ex vivo distribution of the three myeloid cell subsets between pediatric and elderly donors, we found a similar distribution of the myeloid differentiating cells (Fig. 1a, left panels; Fig. 1b), suggesting that there is no significant change in their distribution with aging, despite a reported shift toward myelopoiesis [26]. Instead, after 4 days of cell culture in the presence of myelomonocytic cytokines we observed a significant reduction in the percentage of suppressive i-BM-MDSCs in the group of elderly with respect to the pediatric group (median 12.6% vs 8.1%, *p* = 0.0001 by pairwise comparison) (Fig. 1c). Moreover, compared to the freshly isolated BM cells, in which polymorphonuclear (PMN) cells corresponding to CD11b^+^/CD16^high^ are clearly present, after cell culture with the cytokines only a very low presence of these mature cells can be detected, although significantly higher in pediatric donors (median 8.4%, *n* = 20) compared to elderly donors (median 2.4%, *n* = 19) (Fig. 1a left panels; Fig. 1c).

**Fig. 2:**
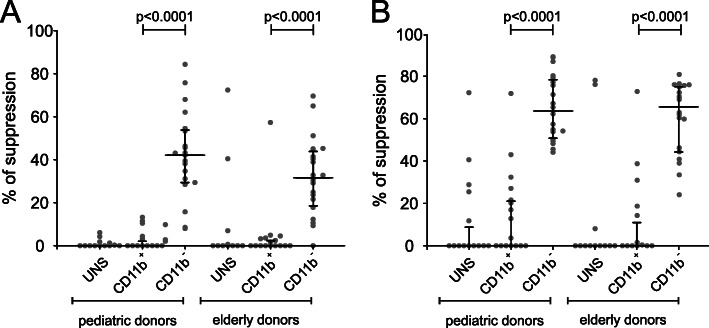
Suppression of PBMCs proliferation by myeloid subsets sorted from BM or BM-MDSCs. CellTrace-labelled PBMCs activated with anti-CD3 and anti-CD28 were cultured in the presence of 1:1 ratio of the myeloid populations, unsorted (UNS), mature (CD11b^+^) or immature (CD11b^−^, BM-MDSCs). Immunosuppression of these populations was evaluated on activated T cells, (gated as CellTrace^+^/CD3^+^) normalized on the control without myeloid cells. (*n* = 20 independent experiments for pediatric patients, *n* = 20 for elderly patients) **a** Suppression was calculated by CellTrace profile assessed as the reduction of the proliferating CD3^+^ cells (those contained within generation two onward) in the co-culture condition as compared to T cells cultured alone, expressed as percentage. **b** Suppression was assessed by T cell number calculated by analyzing the absolute number of proliferating CD3^+^ cells by TruCount™ tubes. In both cases proliferation data were normalized assuming as 100% the proliferation rate of CD3^+^ cells cultured without myeloid cells

To assess whether i-BM-MDSCs isolated from the two groups of donors displayed immune suppression on T cells, after 4 days of cell culture two fractions of BM-MDSCs were isolated, corresponding to the more immature CD11b^−^ and the more differentiated CD11b^+^ cell fraction. A functional assay was set up with allogenic peripheral blood mononuclear cells (PBMCs) activated with anti-CD3 and anti-CD28 for 4 days in the presence of the two myeloid cell fractions. Previous work from our laboratory already demonstrated that the CD11b^−^ fraction corresponds to the suppressive i-BM-MDSC, while CD11b^+^ cells are devoid of significant immune suppressive activity [19]. Evaluation of the proliferation of T cells was assessed by CellTrace profile (Fig. 2), as previously described [27]. In line with previous results, only the most immature CD11b^−^ cell fraction showed the ability to suppress the proliferation of T cells in both groups of donors (Fig. 2 a-b) [19] and, importantly, no significant differences were found comparing the suppression ability of CD11b^−^BM-MDSCs isolated from pediatric and elderly donors, thus suggesting that the suppressive ability of MDSCs is maintained with aging. Of note, the immune suppressive capacity is acquired only after cytokines induction, as demonstrated by functional tests performed on ex vivo sorted immature CD11b^−^ cells from the BM of both the pediatric [19] and elderly (data not shown) donors, that failed to suppress T cell proliferation.

We previously demonstrated that MDSCs can be induced in vitro by using a combination of cytokines and that CD11b^−^/CD16^−^ cells phenotypically and functionally resembled to myeloid suppressor cells present in the blood of cancer patients [19, 25]. In the present work, we extend these data and observe that a reduced percentage of immune suppressive myeloid cells can be generated with G-CSF and GM-CSF combination from the BM of elderly donors, suggesting a less effective ability to induce this mechanism. Of note, the reduced frequency of BM-MDSCs in elderly donors does not translate in a worsened MDSCs function, since this population maintains the same immunosuppressive capacity of pediatric donors, as demonstrated by the elevated suppression observed in a functional assay. Literature data report that the aging process is associated with a significant increase in MDSCs [23], and accompanied by an augmented myeloid-to-lymphoid ratio [26], although it was never clarified the mechanism of this age-related process. Actually, conflicting results exist concerning the cellular complexity of the bone marrow niche during aging [28], and our understanding of MDSC generation during aging remains limited [22]. However, our findings suggest that in vitro induction of MDSCs from the BM is reduced with aging and, this may be due to an impaired ability of myeloid precursors to proliferate, as observed in T cells [14], or could be due to an impaired response to cytokine stimuli [29]. Therefore, the increased peripheral MDSC levels observed in cancer patients might be dependent on cancer-associated mechanisms, rather than on the aging process itself.

## Materials and methods

### Patients information

Two series of consecutive patients were recruited in this study. One of these corresponded to elderly who underwent total hip arthroplasty (THA) or hip endoprosthesis at the Orthopedic Clinic, and the second included pediatric patients enrolled in the protocol AIEOP-BFM-ALL 2000, with suspected leukemia or lymphomas, with lymphatic leukemia after 78 days without recurrences, and with lymphatic leukemia after BM transplantation as a part of the diagnostic follow-up. For adult patients one of the inclusion criteria was suspension of steroid therapy for at least 3 months. Exclusion criteria were cancer, infections, sickle cell anemia and autoimmune diseases. Informed consent was obtained from all participants, in compliance with all the relevant national regulations, institutional policies and in accordance the tenets of the Helsinki Declaration, and has been approved by the local Ethic Committee (CESC Code: n. 95).

### BM-MDSC induction and cell subsets separation

Fresh BM aspirates were treated with K_2_EDTA to prevent coagulation, and lysed to remove red blood cells with a hypotonic solution of ammonium chloride. Myeloid populations were isolated through magnetic sorting by the depletion of CD3^+^/CD19^+^/CD56^+^ lymphocytes, with a cocktail of immunomagnetic beads obtained by combining anti–human CD3, CD19, and CD56 beads (Miltenyi Biotec). Cell purity was checked by FACS analysis on forward/side scatter parameters. Subsequently, the CD3^−^/CD19^−^/CD56^−^ fraction was cultured with 40 ng/ml G-CSF and GM-CSF (Miltenyi Biotec) for 4 days at 37 °C, 8% CO_2_ in order to obtain BM-MDSCs, as previously described [19, 25]. On the fourth day, cells were collected and separated into CD11b^−^ and CD11b^+^ fractions with immunomagnetic anti–human CD11b beads (Miltenyi Biotec). The purity of sorted cells was checked by staining both fractions with anti-CD16 (BD Pharmingen) and anti-CD11b (Beckman Coulter) antibodies and analyzing cells by FACS Calibur cytometer (BD Biosciences). All the fractions were obtained with a purity of ≥90%.

### Immunophenotyping analysis by flow cytometry

Cell surface staining for flow cytometry was performed as previously described [19]. Briefly, cells were incubated with FcReceptor (FcR) Blocking Reagent (Miltenyi Biotec) and then labeled for 20 min at 4 °C with monoclonal antibodies (Abs) anti-CD11b (Beckman Coulter) and anti-CD16 (BD Pharmingen). Data acquisition was performed with FACSCalibur (BD Biosciences) and samples were analyzed by FlowJo software (Tree Star Inc).

### Proliferation assay and immune suppression evaluation

Peripheral blood mononuclear cells (PBMCs) were isolated from the peripheral blood of healthy donors by density gradient centrifugation on FicollPaque PLUS (GE Healthcare-Amersham, NJ, USA), as previously described [19]. Briefly, PBMCs were stained with 0.5 μM CellTrace™ Violet Cell Proliferation Kit (Invitrogen, Molecular Probes, MA, USA), according to manufacturer’s instructions. CellTrace-labelled PBMCs were activated with coated 1 μg/ml anti-CD3 and 5 μg/ml soluble anti-CD28 (BioLegend) for 4 days and co-cultured in flat bottom 96 well plates at the 1:1 ratio with CD11b^−^, CD11b^+^ and unsorted fractions. Cell cultures were incubated at 37 °C and 5% CO_2_ in arginine free-RPMI (Biological Industries), supplemented with 150 μM arginine, 10% FBS (Sigma-Aldrich), 10 U/ml penicillin and streptomycin, and 10 mM HEPES. After 4 days, cells were harvested, stained with anti-CD3 (Beckman Coulter) and analyzed by flow cytometry. Proliferation of T cells was evaluated by assessing the signal of CellTrace on CD3^+^ cells, and considering as proliferating the cells present from generation 2 onwards, or by calculating the absolute number of CD3^+^/CellTrace^+^ cells in each sample by BD TruCount™ tubes (BD Biosciences). In both cases data were normalized assuming the proliferation of T cells cultured alone as 100%.

### Statistical analysis

The Shapiro-Wilk test was used to determine whether data were distributed normally. Chi-square (χ2) test was performed to compare dichotomous data. Mann-Whitney test or unpaired t-test were used to compare continuous variables. Continuous variables were reported as median ± interquartile range (IQR). A *p* < 0.05 was considered as statistically significant. All analyses were performed with Graphpad Prism version 6.01 or SPSS version 23.0.

## Data Availability

The datasets used and/or analyzed during the current study are available from the corresponding author on reasonable request.

## Abbreviations

BM: Bone marrow
BM-MDSCs: Bone marrow-myeloid-derived suppressor cells
MDSCs: Myeloid-derived suppressor cells
PBMC: Peripheral blood mononuclear cell
FBS: Fetal bovine serum;=
GM-CSF: Granulocyte-macrophage colony stimulating factor
G-CSF: Granulocyte colony stimulating factor
