# Correction to: Human MDSCs derived from the bone marrow maintain their functional ability but have a reduced frequency of induction in the elderly compared to pediatric donors

**DOI:** 10.1186/s12979-020-00209-6

**Published:** 2020-11-25

**Authors:** Sara Magri, Elena Masetto, Samantha Solito, Samuela Francescato, Elisa Belluzzi, Assunta Pozzuoli, Antonio Berizzi, Pietro Ruggieri, Susanna Mandruzzato

**Affiliations:** 1 Department of Surgery, Oncology and Gastroenterology, University of Padova, Via Gattamelata, 64, 35128 Padova, Italy; 2 Veneto Institute of Oncology IOV-IRCCS, Via Gattamelata, 64, 35128 Padova, Italy; 3 Present address: University of Verona, Verona, Italy; 4 Pediatric Onco-Hematology Unit, Department of Women’s and Children’s Health, University of Padova, Padova, Italy; 5 Orthopedic and Traumatologic Clinic, Azienda Ospedaliera di Padova, Padova, Italy

**Correction to: Immun Ageing 17, 27 (2020)**

**https://doi.org/10.1186/s12979-020-00199-5**

Following publication of the original article [1], the authors reported an error in affiliation 2. The correct affiliation 2 is presented below.

“Veneto Institute of Oncology IOV-IRCCS, Via Gattamelata, 64, 35128 Padova, Italy”

The original article has been corrected.
